# A Computational Modeling Approach for Investigating Soft Tissue Balancing in Bicruciate Retaining Knee Arthroplasty

**DOI:** 10.1155/2012/652865

**Published:** 2012-10-02

**Authors:** Shahram Amiri, David R. Wilson

**Affiliations:** ^1^Department of Orthopaedics, University of British Columbia, Vancouver, BC, Canada V5Z 1M9; ^2^Centre for Hip Health and Mobility, Robert H. N. Ho Research Centre, 799-2635 Laurel Street, Vancouver, BC, Canada V5Z 1M9

## Abstract

Bicruciate retaining knee arthroplasty, although has shown improved functions and patient satisfaction compared to other designs of total knee replacement, remains a technically demanding option for treating severe cases of arthritic knees. One of the main challenges in bicruciate retaining arthroplasty is proper balancing of the soft tissue during the surgery. In this study biomechanics of soft tissue balancing was investigated using a validated computational model of the knee joint with high fidelity definitions of the soft tissue structures along with a Taguchi method for design of experiments. The model was used to simulate intraoperative balancing of soft tissue structures following the combinations suggested by an orthogonal array design. The results were used to quantify the corresponding effects on the laxity of the joint under anterior-posterior, internal-external, and varus-valgus loads. These effects were ranked for each ligament bundle to identify the components of laxity which were most sensitive to the corresponding surgical modifications. The resulting map of sensitivity for all the ligament bundles determined the components of laxity most suitable for examination during intraoperative balancing of the soft tissue. Ultimately, a sequence for intraoperative soft tissue balancing was suggested for a bicruciate retaining knee arthroplasty.

## 1. Introduction

Total knee arthroplasty (TKA) is a standard of care for end-stage knee arthritis, in which the diseased surfaces of the knee joint are replaced with prosthesis in order to eliminate pain and restore mobility. Proper functioning of the TKA requires restoration of the anatomical joint lines and adequate balancing of tensions of the remaining soft tissue structures. Adjusting the soft tissue constraints in a TKA is a challenging technical task especially for particular designs of prosthesis that allow preservation of the cruciate ligaments. 

Current soft tissue balancing techniques for the ACL-sacrificing designs of total knee replacement are based on providing equal rectangular extension and flexion space for creating constant and uniform tensions on the medial and collateral ligaments (MCLs and LCLs) [1]. Using blocks and spacers, or even the implants themselves, coupled with stress testing, is simple, quick, and always available for soft tissue balancing. This method is, unfortunately, rather imprecise, very subjective, and particularly difficult to use accurately in flexion due to the rotational freedom of the hip joint [2]. To assist ligament balancing in TKA, several devices are available, including tensors [3–5], spacers [4], and electric instruments [6, 7] as described in previous papers. However, all the current methods for balancing the ligament tensions are focused on only the frontal plane for creating the desired varus-valgus stability and producing an even distribution of the compressive forces on the medial and lateral compartments of the joint. Laxity of the joint, defined as the magnitude of displacement or rotation under an applied force or rotational moment applied manually by the surgeon or through a device, can be considered as an indicator of proper or improper soft tissue tensions. Even though soft tissue balancing also directly affects important translational and rotational constraints of the joint in the anterior-posterior (AP) and internal-external (IE) directions, especially when the cruciate ligaments are preserved, an appropriate procedure for fine-tuning the ligament tensions by taking into consideration all components of the joint laxity has not been investigated. 

The bicruciate retaining knee arthroplasties preserve both of the cruciate ligaments and have theoretical benefits over other designs for improved kinematics, range of motion, patient satisfaction, and the general “feel” of a normal knee [8–12]. Particularly for cases of knees with suboptimal integrity of the cruciate ligaments, recent prosthesis design can potentially take advantage of the remaining soft tissues for more improved functions [13]. Cruciate retaining prostheses also preserve more bone stock (that can be used for revision surgery), and unlike the PCL retaining prostheses they do not produce the unfavorable paradoxical motions [14, 15]. Being a technically demanding procedure, in spite of all the potential advantages, the bicruciate retaining option has not been favorable, even for cases when patients have cruciate ligaments with good functional integrity [16]. Retaining both of the cruciate ligaments by preserving their insertion on the tibia can limit the exposure and subsequently make the procedure more difficult. The procedure can be even more challenging if bone deformities have to be corrected during the operation. The joint lines must be anatomically restored and soft tissues should be balanced correspondingly in order to reestablish stability and balance while avoiding abnormal tensioning of the retained ligaments.

A systematic procedure that can successfully adjust soft tissue tensions in a bicruciate retaining knee replacement can be largely useful for improving TKA outcomes through preservation of native soft tissue structures. Coming up with such procedure requires thorough understanding of the complex relationships between soft tissue tensions and their effects on joint laxity and stability. To help investigate possible soft tissue balancing scenarios, a computational model of the knee joint can provide the necessary means for simulating surgical modifications in the soft tissue structures. For computational models of the knee joint, there is a broad range of options in terms of the choice of the numerical solver and also the fidelity of the shape and mechanical properties of the various structural elements. A rigid body dynamics model of the knee joint with rigid articular surfaces and multiple-bundle spring element representing the ligamentous structures [17] can be a proper choice for studying the effects of structural changes to the joint laxity, especially if the simulation has to be reasonably fast to simulate hundreds of different loading scenarios. When such a model is used along with the methods for design of experiments (DOE) namely the Taguchi's method [18], the impacts of changes in the soft tissue tensions can be quantified systematically. Following Taguchi's approach, discrete values (levels) are assigned to each model parameter and the corresponding effects of each parameter on joint laxity are assessed by conducting simulations according to the selected design of an orthogonal array [18]. The use of fractional factorial design of orthogonal arrays enables assessment of sensitivity of a system to a large number of input parameters, while reducing the experimental effort. Over the traditional method of changing one parameter at a time, the advantage of this approach is that the potential problems associated with selection of a single “base line” condition can be avoided [19]. This is particularly an important point in modeling biological systems that inherently have large parameter variabilities.

The objective of this study was to use a validated computational model of the knee joint with high fidelity ligament definitions, along with a design of experiments approach, to evaluate the effects of variations in soft tissue tensions on the anterior-posterior, internal-external, and varus-valgus laxity characteristics of the knee joint. The findings were used to suggest a sequential plan for systematic balancing of soft tissue tensions in a bicruciate retaining total knee replacement.

## 2. Materials and Methods

### 2.1. Articular Geometry

In a bicruciate retaining design of total knee replacement, in comparison to cruciate sacrificed prostheses, the design of the articular geometry can be closer to the natural anatomy, relying on the retained soft tissues (in ideal well-balanced conditions) to contribute in stability and kinematics similar to a natural knee joint [20]. Based on this hypothesis, in this study the shape of the implant surfaces was constructed based on articular geometry of the natural knee joint. This led to a more congruent medial compartment to provide stability and a less congruent lateral compartment to promote physiological anterior-posterior shift and internal-external pivoting of the lateral compartment. This resemblance to the natural configuration in theory can help restore the physiological functions of the knee joint [21]. For the purpose of model construction, the articular surface of the femur was extracted from cartilage surface geometry. For the tibial articular surface, the shape of the articulation was constructed as a combination of the tibial and meniscal surfaces. The menisci were modeled by merging the anterior and posterior horns of the menisci at their terminal points of motion at extension and full flexion angles, following the details described in previous literature [20]. The patella was not included in the modeling, since during surgery the knee cap is flipped over to one side for exposure of the joint, and the patella is not expected to contribute to the passive laxity of the joint in absence of the quadriceps forces in the intraoperative situation.

### 2.2. Model Construction

**Figure 1:**
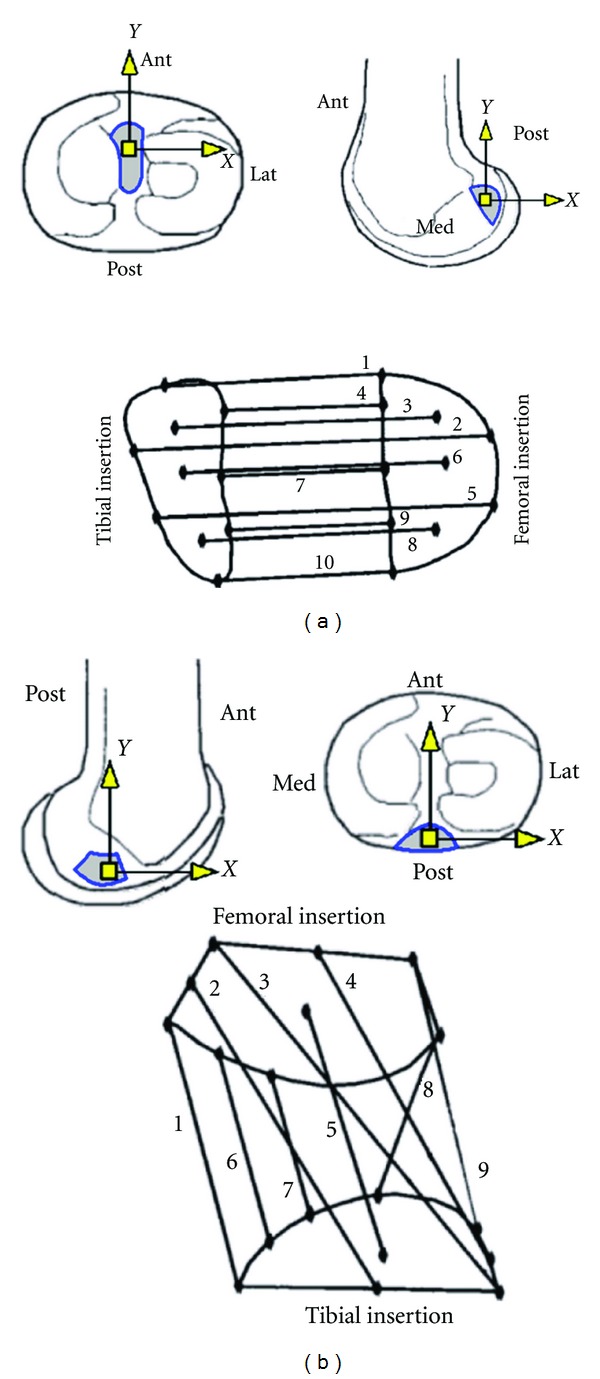
Connectivity maps of the bundles of the ACL (a) and the PCL (b) [22, 23]. The tibial and femoral 2D coordinate systems were identified for each attachment. Locations of the attachments of the ligament bundles were defined with respect to these coordinate systems and on their corresponding *X*-*Y* planes.

**Figure 2:**
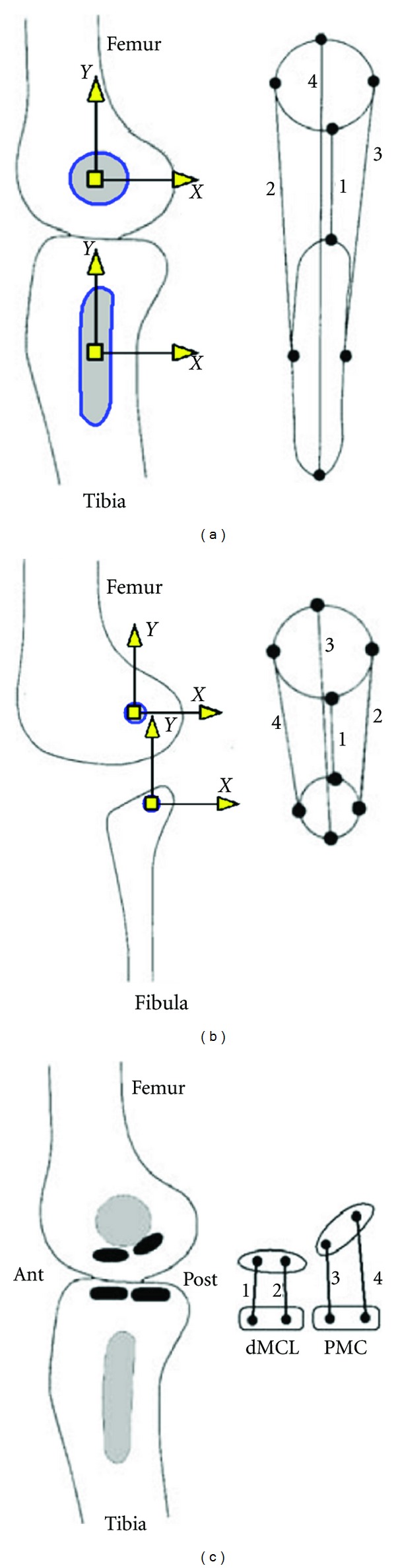
Connectivity maps of the bundles of the superficial bundles of the MCL (sMCL) (a), the lateral collateral ligament (LCL) (b) [23, 24], and the deep bundles of the MCL and the posteromedial capsule (PMC) (c) [25]. The tibial and femoral coordinate system was identified for each attachment. Locations of the attachments of the ligament bundles were defined with respect to these coordinate systems and on their corresponding *X*-*Y* plane.

A validated computer model of the knee joint, previously constructed to investigate the biomechanical functions [20], was utilized for the purpose of this study. The model used in this study was previously validated against patterns of motion of the joint and the experimental measures of the ligament tensions [20, 21]. The following provides the description of the elements and construction of the model. The geometry of the model for the shapes of the articular surfaces and the attachment sites of the ligaments were obtained from laser scanning of a dissected cadaveric specimen. The surface geometries of the articular surfaces of the tibia and femur were generated as stereolithography (STL) mesh files in IMInspect Version 8.0 (InnovMetric, Quebec, QC, Canada). MSC.ADAMS/View 2003 (MSC.Software, Santa Ana, CA) was used to construct the model and to simulate the laxity of the joint at multiple flexion angles under various external loads. Six main ligament groups were considered including the anterior cruciate ligament (ACL), the posterior cruciate ligament (PCL), the lateral collateral ligament (LCL), the superficial and deep bundles of the medial collateral ligament (sMCL and dMCL), and the posteromedial capsule (PMC). To determine the attachment locations of the origin and insertion of the individual ligament bundles, the bundle connectivity maps of the attachment sites of the ligaments reported in the literature [22, 23, 26, 27] were scaled to the digitized footprints of the ligament attachments (Figures 1 and 2). The ACL and PCL were modeled with 10 and 9 spring elements, respectively, to better capture the complex kinematics of the ligaments [17] compared to the two-bundle models used in some of the analytical studies [28, 29]. The sMCL and LCL were modeled with 4 bundles, and the dMCL and PMC were modeled with 2 bundles each (Figure 2). A nonlinear force-displacement relationship (1) to (5) was used to define the deformation of the spring elements as described in the literature [28]. In these equations, “*f*” is the ligament tension, “*ε*” is the ligament strain, “*k*” is a stiffness parameter,“*c*” is a constant term (=0.03), “*L*” is the length of the ligament and “*L*_0_” is zero length of the ligament, “*L*_*i*_” is the ligament length at full extension, and the reference strain “*ε*_*i*_” is the corresponding strain at full extension:

(1) f(ε)=14kε2c when  0≤ε≤2c,

(2) f(ε)=k(ε−c) when  ε>2c,

(3) f(ε)=0 when  ε<0,

(4) ε=(L−L0)L0,

(5) εi=(Li−L0)L0.

**Figure 3:**
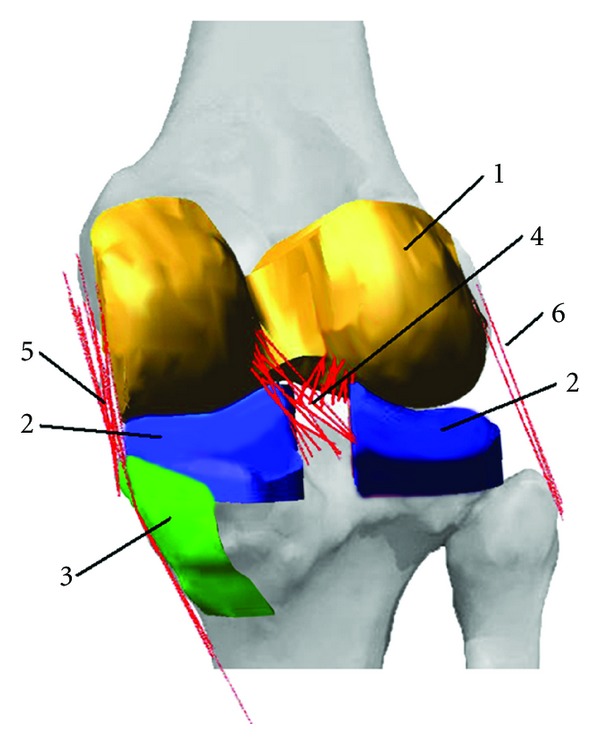
A view of the computational model of the knee joint was constructed in MSC.ADAMS/View. The model consists of (1 and 2) the geometries of the articular surfaces of the femur and the tibia, (3) the surface of the medial aspect of the tibia that interacted with the MCL to simulate wrapping of the bundles, (4) the anterior and posterior cruciate ligaments, (5) the MCL and PMC, and (6) the LCL (for ligament name abbreviations refer to description of Table 1.)

The stiffness characteristic of each ligament group (*k*) was set as the average values found in the literature [28, 30–32], assuming that stiffness was equally distributed among all bundles within a ligament division. The reference strains of the ligament bundles (*ε*_*i*_) were selected from the literature [32, 33]. For the ACL and PCL, the tibial attachments of the ligaments were divided into anterior, posterior, and middle regions. For the sMCL, dMCL, LCL, and PMC, the ligaments were divided into the anterior and posterior subregions. The reference strains of the ligament bundles located within each of these sections were given the same values. The selected values for the model parameters are listed in Table 1. To model the wrapping effects of the MCL around the edge of the tibial plateau [34], spheres of 1 mm in diameter were attached to the midpoint of the MCL spring elements of the model, and rigid contacts were defined between these spheres and the medial aspect of the tibial bone. Diameter of 1 mm for the spheres was determined by trial and error as appropriate size for detecting contact between the corresponding surfaces in the simulations. A view of the model constructed in MSC.ADAMS/View is shown in Figure 3.

### 2.3. Model Parameters

To investigate the effects of variations of the soft tissue structures on the laxity and stability characteristics of the joint, the ligament bundles were defined parametrically. A MATLAB computer program (MathWorks, Natick, MA, USA) was developed to generate MSC.ADAMS/View Script for changing the model parameters, running the simulations, and extracting the kinematics results. The intraoperative modifications in the soft tissue tensions were simulated by changing the reference strains of the ligaments in the model. To account for uncertainties in the locations of the attachment sites and the mechanical properties of the ligaments in the analysis, the ligament attachments and stiffness of the ligaments were also defined parametrically.

For each ligament attachment, a 2D *XY* coordinate system was defined on a flat plane fitted to the corresponding attachment site, with its origin at the centroid of the attachment area (Figures 1 and 2). Changes made to the attachment location of one ligament were applied simultaneously to all bundles associated with that ligament with respect to its 2D coordinate system. In total, 40 parameters were used to model the 6 groups of ligaments. For each ligament, 4 parameters defined the attachment locations on the tibia (*TX*, *TY*) and femur (*FX*, *FY*) (parameters 1 to 20 in Table 2). When the location of an attachment of a ligament was modified, the length of the bundle in the force-displacement equations (4) was updated in order to keep the ligament strain unaffected. Six parameters were used to define the stiffness (*k*) of different ligaments (parameters 20 to 26 in Table 2). The reference strains of the ligament bundle regions were defined by 14 parameters (parameters 27 to 40 in Table 2). The ACL and PCL ligament bundles were grouped into three different sections (anterior, middle, and posterior), and the sMCL, dMCL, and PMC were grouped into two different (anterior and posterior) regions.

### 2.4. Simulating the Laxity Tests

**Figure 4:**
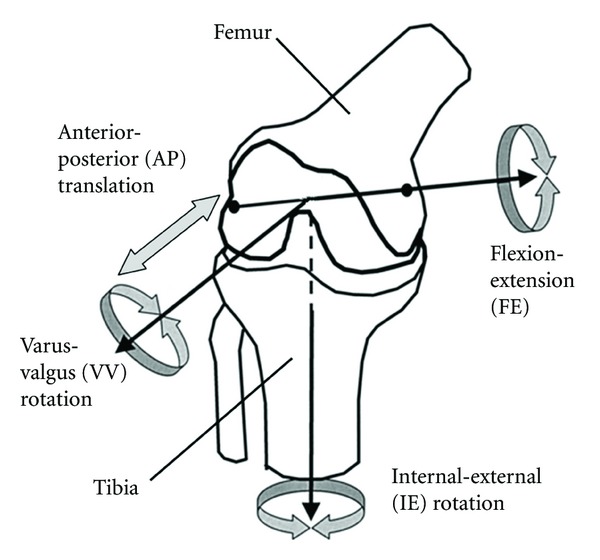
Directions of the rotations and translations according to Grood and Suntay's coordinate system definition [35].

The model was run in quasistatic mode with dynamic equilibrium option in MSC.ADAMS/View. Rigid-frictionless contacts were defined between the articular surfaces of the tibia and femur, assuming that friction has minimal effects under very light compressive forces during intraoperative laxity assessments. Grood and Suntay's convention was used to define the joint coordinates system [35] (Figure 4), and the anterior-posterior, internal-external, and varus-valgus laxities were defined as the total magnitude of translation or rotation of the joint under the corresponding external loads [36]. These loads simulate the intraoperative scenarios where the surgeon applies loads to the joint either manually or uses a device to assess the laxity and stability of the joint.

Each simulation began with the knee at zero flexion and the femur in a fixed position, and a sinusoidal drawer force with amplitude of 100 N applied along the anterior-posterior axis of the joint and to the centre of the tibial plateau. The internal-external laxity test was simulated next by applying an 8 Nm amplitude sinusoidal moment to the tibia and about its long axis. The simulation continued by a varus-valgus laxity test through applying a 30 Nm amplitude sinusoidal moment to the tibia and about the anterior-posterior axis of the joint. During each laxity test, only the flexion angle was kept fixed and the rest of the degrees of freedom were left unconstrained. The flexion angle of the joint was then incrementally increased by 30 degrees and all three laxity tests described above were repeated at the new joint position. The simulation was repeated for 60°, 90°, and 120° of flexion. The output of the model in terms of the response displacements along and about the clinical axes of the joint was automatically extracted and analyzed using a program developed in MATLAB programming language.

### 2.5. Matrix of Simulations

Design of experiments (DOE) was conducted based on 40 parameters defining all aspects of the ligament bundles in the model, including their stiffness, attachment locations, and reference strains. The Taguchi standard orthogonal array L81(3^40^) was used to investigate the combined effects of the 40 model parameters at 3 levels, by conducting 81 simulation runs [18]. The selected experimental design permitted study of the main factors, assuming synergistic interactions among the model parameters [18]. The three levels of variations for the parameters that defined the reference strains of the ligaments were set as their nominal and at ±0.05 perturbations. A 0.05 change in the strain of a ligament bundle of an average 20 mm in length corresponds to a 1 mm change in the length of the ligament, which was assumed to be a reasonable controllable length during intraoperative release of a ligament bundle. In order to account for sensitivity of the model to the attachment sites of the ligaments and the stiffness values, perturbations of ±1 mm and ±5% were considered, respectively, assuming that these are reasonable margins of uncertainty for these parameters. The values of the three levels of the model parameters used in the design of the matrix of experiments are listed in Table 2. With these values the model was stable numerically and there were no model convergence issues for any combination of the parameters. Analysis of variance (ANOVA) was conducted to determine the main effect of each parameter on the laxity of the joint. For every laxity test, the relative importance (RIM) of each model parameter was calculated using (6) to (8):

(6) $$\begin{array}{c} {\text{RIM}}_{A}=\frac{{\text{SS}}_{A}}{\text{TSS}}\;“\text{Relative}\;\;\text{Importance”}\;\text{of}\;\text{the}\;\text{parameter}\;A, \end{array}$$

(7) SSA=27·(mA1−m)2+27·(mA2−m)2+27·(mA3−m)2“Sum  of  squares” due  to  parameter  A,

(8) TSS=∑i=181(ηi−m)2, where  m=∑i=181ηi81“Total  sum  of  squares”,ηi:  “magnitude  of  the  laxity  of  the  joint”for  the  ith  rows  of  the  matrix  of  experiment.

In these equations *m*_*A*1_, *m*_*A*2_, and *m*_*A*3_ are the mean laxity measures for the three different levels of the parameter “*A*” in the orthogonal array. Number 27 in (7) corresponds with the number of times each level of parameter “*A*” was repeated in the rows of the matrix of experiments. The ratio of sum of squares over the total sum of squares (5) estimates the approximate contribution of each parameter and is a measure of its relative importance [37]. Parameters with higher relative importance (RIM) are those which if changed, they would cause larger magnitude of effects on the corresponding component of the joint laxity. In total 1215 laxity simulations (81∗5∗3 = 1215) were run to assess the relative importance of all model parameters corresponding to all components of laxity (anterior-posterior, internal-external, and varus-valgus) for 30° intervals of flexion angle (0°, 30°, 60°, 90°, and 120°). 

## 3. Results 

**Figure 5:**
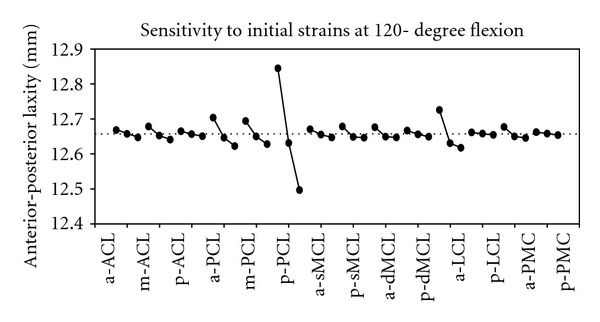
Effects of the changes in the reference strains of various ligament bundles on the anterior-posterior (AP) laxity of the joint at 120° flexion. The mean value of the laxity has been shown as a dotted line. The posterior bundles of the PCL and the anterior bundles of the LCL were found to have the highest impacts. (Abbreviations: a-: anterior bundles; m-: middle bundles; p-: posterior bundles. Other abbreviations are described in Tables 1 and 3. For ligament name abbreviations refer to the description of Table 1.)

As a visual illustration of the effects of perturbations on various components of laxity in an example, Figure 5 graphically makes the comparison among model parameters in terms of their effects on joint laxity in the anterior-posterior direction at 120°. Tensions in the posterior bundles of the PCL and the anterior bundles of the LCL had the most effects on the anterior-posterior laxity of the joint at 120° flexion, as graphically shown in this example plot (Figure 5), and their corresponding RIM can indicate (5). The relative importance factors (RIM) of all parameters defining the reference strains of the ligaments in terms of their effects on various components of the joint laxity are summarized in Table 3. For each group of bundles, the corresponding RIMs were compared among all components of laxity and linearly scales and ranked from 1 to 5. The cells of the table with higher ranks and darker color corresponded to the components of laxity most affected by changes made to the tensions of particular ligament bundles (Table 3). 

The results were also rearranged and summarized to identify the most sensitive laxity tests for each ligament of the knee joint (Table 4). The average of the rankings of the relative importance of the bundles in each ligament identified the overall most sensitive laxity tests. The laxity tests with the average relative importance (RIM) of equal or greater than 3 were selected as appropriate modes of intraoperative examination for soft tissue adjustments (Table 4). The anterior-posterior component of laxity at 0° and 120°, the internal-external component of the laxity at 30°, 60°, and 120°, and the varus-valgus component of laxity at 0° and 30° were most sensitive to the variations in the ligament strains.

## 4. Discussion

Correct balancing of soft tissues is critical for successful total knee arthroplasty procedures. Release and retension of the ligaments are normally conducted during knee arthroplasty surgery to avoid abnormal pulls of the soft tissue and to produce even distribution of the contact forces on the medial and lateral sides of the joint throughout the range of motion. This task is challenging for the PCL retaining types of the prosthesis and even more technically demanding for bicruciate retaining designs of the implant. This study investigated the effects of variations in the ligament strains on multiple components of laxity of the knee joint. The results suggest a sequence for balancing the soft tissue tensions in bicruciate retaining knee arthroplasty.

Most of the published experimental and analytical reports are focused on specific ligaments and laxity under particular loading conditions, and there is not enough data in the literature for comparison with all the results found in this study. However, in terms of effects of the reference strains of the ligament on the joint laxity, there is consistency between the results found in this study and the literature. Fleming et al. investigated the effects of tension of the ACL prosthesis on the AP laxity. Their report shows that the AP laxity is sensitive to the tension of the ACL ligament in early flexion [38]. If changing the reference strain of a ligament under tension is assumed synonymous to changing the magnitude of the ligament tension, agreement can be observed between the findings of Fleming et al. and the high relative importance values found in this study for the posterior and middle bundles of the ACL at 0° flexion (Table 3).

The results of this study indicate that different laxity tests and flexion angles might be necessary for optimal balancing of the soft tissue constraints of the knee joint during knee arthroplasty. The selection of ligaments with the most impact on laxity (cells with larger RIM values and darker colors in Table 3) varied substantially for different directions of motion and different flexion angles. This was seen even among different fiber bundles within the same ligament, which can be related to different recruitment patterns imposed to the bundles. For instance, the anterior-posterior laxity of the joint at 0° (AP-00) is not greatly influenced by changes in the strains of the anterior bundles of the ACL (RIM = 2), as much as it is affected by changes in the strains of the middle and posterior bundles (RIM = 4). As another example, considering the anterior-posterior laxity tests, the highest ranks of the relative importance for the anterior bundles of the PCL were at 30° and 90° of flexion (AP-30 and AP-90), whereas for the middle and posterior bundles the corresponding high sensitivity was observed at 0° and 120°, respectively (AP-00 and AP-120). In some cases, the joint laxity under a particular external load was uniformly affected by variations in strains of all the ligament bundles. For instance, the varus-valgus laxity of the joint at 0° (VV-00) was affected by change in the strains of the anterior, middle, and posterior bundles of the ACL and PCL. 

The map of sensitivity provided for multiple laxity components of the knee joint (Table 3) highlights some of the components of the laxity that are leading candidates for intraoperative examination during knee arthroplasty. Many of the laxity tests were not identified with high RIM ranking (RIM > 3) for any particular ligament bundle (e.g., AP-60 and VV-90) (Table 3). On the other hand, some of the laxity tests were sensitive to strains of multiple ligament bundles: the varus-valgus laxity at 0° flexion (VV-00) had the highest sensitivity rank (RIM = 5) for all the ligament bundles, except for the posterior bundles of the dMCL and the PMC. The internal-external laxity at 120° (IE-120) also had the highest sensitivity rank for most of the superficial and deep bundles of the MCL. These highlight the importance of incorporating these laxity tests (VV-00 and IE-120) in the soft tissue balancing process. In total, 8 laxity tests were identified with the highest sensitivity to the ligament strains (Table 4). For the ACL only two laxities stood out with high RIM (AP-00 and VV-00). On the other hand, the LCL was highly sensitive to multiple components of laxity in different planes and flexion angles (Table 4), which is expected to be related to the geometric location of the LCL on the lateral side of the joint and more mobility of the lateral compartment under various applied forces and moments.

Based on the findings of this study (Tables 3 and 4) there is an optimal sequence for adjusting strains of 14 ligament bundles during surgery using the 8 selected laxity examinations. The suggested plan for soft tissue balancing is illustrated in Table 5. Going through the steps from the top to the bottom of the table, the number of the unadjusted ligament tensions reduces step by step until all the ligaments are balanced. The strategy in planning the sequence lies in gradually reducing the complexity starting from the components of laxity that are sensitive to only a smaller number of ligament bundles (i.e., AP-00 and IE-00), so that for the next steps in the sequence the number of the unadjusted ligaments are reduced for the tests that are sensitive to a larger number of ligament bundles (IE-120 and VV-00). 

A strength of the study is that it uses a novel computational modeling approach for investigating the biomechanical effects of the soft tissue strains on laxity of the knee joint. The use of the design of experiment method allowed assessment of the effects of 40 model parameters based on only 81 observations. It would have not been practical to consider full-factorial combination of the model parameters, which requires 3^40^ observations for 40 model parameters at 3 levels. For studying the contributions of the ligaments to the joint stability and kinematics, typically in vitro studies are conducted on cadaver specimens using robotics manipulators [39]. However, controlling the exact soft strains while maintaining the other factors unchanged in the described design of experiments is only practical in a numerical modeling scheme.

One limitation of the study is that the geometry of commercially available bicruciate retaining knee prostheses was not included in the model. However, since bicruciate knee prostheses have geometric congruency similar to the articular surfaces of the natural knee joint, having a model with a natural articular geometry is justified. The patella was not included in the modeling, which is a reasonable assumption, considering that in the intraoperative ligament balancing, the knee cap is expected to be flipped over on a side and therefore not contributing to the laxity of the joint. Furthermore, the effects of differences in the shapes of the bones and articular surface morphology were not considered in this study, which can be justified considering that the focus of the study was on investigating an optimal soft tissue balancing technique in a model that represents an average natural knee geometry. It is also important to note that this study was focused on release of soft tissue as one of the most important means for adjusting the balance of soft tissues. However, other factors including component design and size, component position and alignments, osteophyte resection, and bone resection all can have direct effects on tightness or looseness of the soft tissues and therefore can be included in the soft tissue balancing process.

Another limitation is that the suggested sequence for adjusting the ligament bundles and its effectiveness were not tested on cadaveric specimens. Such a test would require techniques for intraoperative assessment of the joint laxity along and about three axes (anterior-posterior, internal-external, and varus-valgus), for which an adequate surgical tool is not currently available. Current soft tissue balancing instruments are designed for only adjusting varus-valgus laxities of the joint in the frontal plane. These tools can be sufficient for when both of the cruciate ligaments are sacrificed, but they cannot provide the required 3-axes measurements for bicruciate retaining prostheses as discussed in this study. Implementation of the suggested sequence requires developing more advanced tools, perhaps in combination with a computer assisted navigation system [40], for accurate multiple-axes examination and balancing of soft tissue constraints. 

## 5. Conclusions

In this study a computational modeling approach was used to identify the most sensitive components of knee joint laxity and to suggest a sequence for balancing ligament tensions during knee arthroplasty when both of the cruciate ligaments are preserved. Findings of this study highlights the importance of considering multiple degrees of freedom in balancing soft tissues during knee arthroplasty, and motivates development of new surgical tools for overcoming the current technical challenges with bircruciate retaining knee prosthesis.

## Figures and Tables

**Table 1:** Ligament bundle definition and the values assigned to their stiffness and reference strains in the computational model of the knee joint [32, 33]. (Abbreviations: ACL: anterior cruciate ligament; PCL: posterior cruciate ligament; sMCL: superior bundles of the medial collateral ligament; dMCL: deep bundles of the medial collateral ligament; LCL: lateral collateral ligament; PMC: posterior-medial capsule).

Ligament	ACL			PCL			sMCL		dMCL	LCL		PMC
bundle group	Anterior	Middle	Posterior	Anterior	Middle	Posterior	Anterior	Posterior	Anterior and posterior	Anterior	Posterior	Anterior and posterior
Number of bundles	4	3	3	3	3	3	2	2	2	2	2	2
Stiffness (*N*)	5000			9000			2750		1000	2000		1000
Initial strain (ε_*i*_)	0.160	0.100	0.100	−0.068	−0.169	−0.169	0.180	0.180	0.030	0.050	0.050	0.030

**Table 2:** List of the model parameters and the corresponding three levels used in the design of experiments. Parameters 1 to 20 define deviations of the coordinates of the ligament attachments (in mm) on the tibia (*TX*, *TY*) and the femur (*FX*, *FY*) with respect to their corresponding 2D coordinate system (Figures 1 and 2). Parameters 21 to 25 define the stiffness parameters (*K*) for the six different ligaments (in *N*). Parameters 27 to 40 are assigned to the reference strains of the ligament bundles (*ε*). (For the ligament name abbreviations see the description of Table 1.)

Parameter no.	1	2	3	4	5	6	7	8	9	10	11	12	13	14	15	16	17	18	19	20
Ligament	ACL	ACL	ACL	ACL	PCL	PCL	PCL	PCL	sMCL	sMCL	sMCL	sMCL	LCL	LCL	LCL	LCL	dMCL and PMC			
Parameter name	TX	TY	FX	FY	TX	TY	FX	FY	TX	TY	FX	FY	TX	TY	FX	FY	TX	TY	FX	FY
Level #1	−1	−1	−1	−1	−1	−1	−1	−1	−1	−1	−1	−1	−1	−1	−1	−1	−1	−1	−1	−1
Level #2	0	0	0	0	0	0	0	0	0	0	0	0	0	0	0	0	0	0	0	0
Level #3	1	1	1	1	1	1	1	1	1	1	1	1	1	1	1	1	1	1	1	1

Parameter #	21	22	23	24	25	26	27	28	29	30	31	32	33	34	35	36	37	38	39	40

Ligament	ACL	PCL	sMCL	dMCL	LCL	PMC	ACL	ACL	ACL	PCL	PCL	PCL	sMCL	sMCL	dMCL	dMCL	LCL	LCL	PMC	PMC
Group							Anterior	Middle	Posterior	Anterior	Middle	Posterior	Anterior	Posterior	Anterior	Posterior	Anterior	Posterior	Anterior	Posterior
Parameter name	*K*	*K*	*K*	*K*	*K*	*K*	ε_*i*_	ε_*i*_	ε_*i*_	ε_*i*_	ε_*i*_	ε_*i*_	ε_*i*_	ε_*i*_	ε_*i*_	ε_*i*_	ε_*i*_	ε_*i*_	ε_*i*_	ε_*i*_
Level #1	4750	8550	2613	950	2613	950	0.155	0.095	0.095	−0.073	−0.174	−0.174	0.175	0.175	0.025	0.025	0.045	0.045	0.025	0.025
Level #2	5000	9000	2750	1000	2750	1000	0.160	0.100	0.100	−0.068	−0.169	−0.169	0.180	0.180	0.030	0.030	0.050	0.050	0.030	0.030
Level #3	5250	9450	2888	1050	2888	1050	0.165	0.105	0.105	−0.063	−0.164	−0.164	0.185	0.185	0.035	0.035	0.055	0.055	0.035	0.035

**Table 3:** For each individual ligament bundle the values of relative importance (RIM) calculated for various components of laxity were compared and ranked from 1 to 5 in the table. Number 5 and black cells identify the loading condition for which changes in the corresponding ligament bundle have the highest relative importance. Smaller numbers and lighter color cells correspond with smaller RIM. The AP, IE, and VV on the left column refer to the anterior-posterior, internal-external, and varus-valgus axes, and the numbers in front of these letters indicate the corresponding flexion angle (e.g., AP-30: anterior-posterior laxity test at 30° flexion). For ligament name abbreviations refer to the description of Table 1.

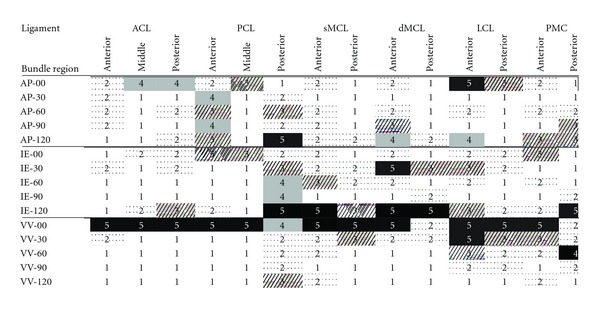

**Table 4:** Ranking of the components of laxity according to sensitivity to the reference strains for individual ligaments (1: the least sensitivity, 5: the highest sensitivity). The most sensitive laxity components (RIM > 2) are chosen on the right column. For definition of the abbreviations used in this table refer to Table 3.

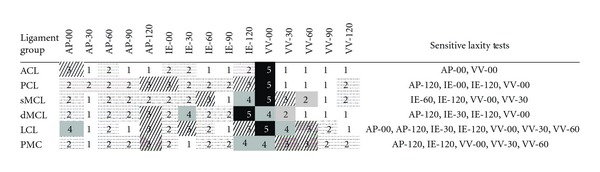

**Table 5:** Suggested sequence for soft tissue balancing in a bicruciate retaining knee arthroplasty and the corresponding laxity tests. Cells with “*” indicate the ligament bundle group that has not been adjusted yet in the process; “X” marks identify the ligament bundles that are suggested to be adjusted in the corresponding step during the sequence. Empty cells correspond with the ligament bundles that have already been adjusted in the previous steps of the sequence. (Abbreviations: a-: anterior bundles; m-: middle bundles; p-: posterior bundles. Other abbreviations are described in Tables 1 and 3).

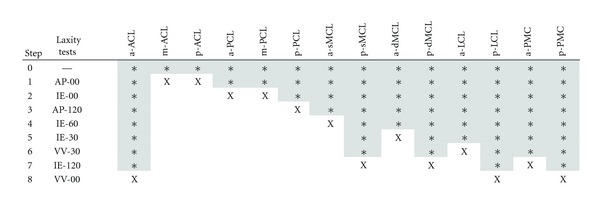

## References

[1] Williams TJ, Thornhill TS, Bellemans J, Ries MD, Victor J (2005). The technique of PCL retention in total knee arthroplasty. *Total Knee Arthroplasty: A Guide To Get Better Performance*.

[2] Wilton TJ, Bellemans J, Ries MD, Victor J (2005). Use of a tensiometer at total knee arthroplasty. *Total Knee Arthroplasty: A Guide to Get Better Performance*.

[3] Laskin RS, Rieger MA (1989). The surgical technique for performing a total knee replacement arthroplasty. *Orthopedic Clinics of North America*.

[4] Sambatakakis A, Attfield SF, Newton G (1993). Quantification of soft-tissue imbalance in condylar knee arthroplasty. *Journal of Biomedical Engineering*.

[5] Winemaker MJ (2002). Perfect balance in total knee arthroplasty: the elusive compromise. *Journal of Arthroplasty*.

[6] Attfield SF, Warren-Forward M, Wilton T, Sambatakakis A (1994). Measurement of soft tissue imbalance in total knee arthroplasty using electronic instrumentation. *Medical Engineering and Physics*.

[7] Morris BA, D’Lima DD, Slamin J (2001). E-Knee: evolution of the electronic knee prosthesis. Telemetry technology development. *Journal of Bone and Joint Surgery*.

[8] Catani F, Ensini A, Belvedere C (2009). In vivo kinematics and kinetics of a bi-cruciate substituting total knee arthroplasty: a combined fluoroscopic and gait analysis study. *Journal of Orthopaedic Research*.

[9] Moro-Oka TA, Muenchinger M, Canciani JP, Banks SA (2007). Comparing in vivo kinematics of anterior cruciate-retaining and posterior cruciate-retaining total knee arthroplasty. *Knee Surgery, Sports Traumatology, Arthroscopy*.

[10] Stiehl JB, Komistek RD, Cloutier JM, Dennis DA (2000). The cruciate ligaments in total knee arthroplasty: a kinematic analysis of 2 total knee arthroplasties. *Journal of Arthroplasty*.

[11] Cloutier JM, Sabouret P, Deghrar A (1999). Total knee arthroplasty with retention of both cruciate ligaments: a nine to eleven-year follow-up study. *Journal of Bone and Joint Surgery*.

[12] Pritchett JW (1996). Anterior cruciate-retaining total knee arthroplasty. *Journal of Arthroplasty*.

[13] Amiri S, Cooke TDV, Wyss UP (2011). Conceptual design for condylar guiding features of a total knee replacement. *Journal of Medical Devices, Transactions of the ASME*.

[14] Banks SA, Bellemans J, Nozaki H, Whiteside LA, Harman M, Hodge WA (2003). Knee motions during maximum flexion in fixed and mobile-bearing arthroplasties. *Clinical Orthopaedics and Related Research*.

[15] Dennis DA, Komistek RD, Mahfouz MR, Haas BD, Stiehl JB (2003). Multicenter determination of in vivo kinematics after total knee arthroplasty. *Clinical Orthopaedics and Related Research*.

[16] Jacofsky D, Bellemans J, Ries MD, Victor J (2005). Bicruciate-retaining total knee arthroplasty. *Total Knee Arthroplasty: A Guide to Get Better Performance*.

[17] Amiri S, Cooke TDV, Wyss UP (2011). A multiple-bundle model to characterize the mechanical behavior of the cruciate ligaments. *Knee*.

[18] Phadke MS (1989). *Quality Engineering Using Robust Design*.

[19] Yao J, Funkenbusch PD, Snibbe J, Maloney M, Lerner AL (2006). Sensitivities of medial meniscal motion and deformation to material properties of articular cartilage, meniscus and meniscal attachments using design of experiments methods. *Journal of Biomechanical Engineering*.

[20] Amiri S, Cooke D, Kim IY, Wyss U (2007). Mechanics of the passive knee joint. Part 2: interaction between the ligaments and the articular surfaces in guiding the joint motion. *Proceedings of the Institution of Mechanical Engineers, Part H*.

[21] Amiri S, Cooke D, Kim IY, Wyss U (2006). Mechanics of the passive knee joint. Part 1: the role of the tibial articular surfaces in guiding the passive motion. *Proceedings of the Institution of Mechanical Engineers, Part H*.

[22] Mommersteeg TJA, Blankevoort L, Huiskes R, Kooloos JGM, Kauer JMG (1996). Characterization of the mechanical behavior of human knee ligaments: a numerical-experimental approach. *Journal of Biomechanics*.

[23] Feikes J, O'Connor JJ, Gill RHS, Zavatsky AB (2003). *Daniel's Knee Injuries*.

[24] Friederich NF, Muller W, O’Brien WR (1992). Clinical application of biomechanical and functional anatomical data of the knee joint. *Orthopade*.

[25] Robinson JR, Bull AMJ, Amis AA (2005). Structural properties of the medial collateral ligament complex of the human knee. *Journal of Biomechanics*.

[26] Friederich NF, Muller W, O’Brien WR (1992). Clinical application of biomechanical and functional anatomical data of the knee joint. *Orthopade*.

[27] Robinson JR, Bull AMJ, Amis AA (2005). Structural properties of the medial collateral ligament complex of the human knee. *Journal of Biomechanics*.

[28] Blankevoort L, Huiskes R (1996). Validation of a three-dimensional model of the knee. *Journal of Biomechanics*.

[29] Crowninshield R, Pope MH, Johnson RJ (1976). An analytical model of the knee. *Journal of Biomechanics*.

[30] Abdel-Rahman EM, Hefzy MS (1998). Three-dimensional dynamic behaviour of the human knee joint under impact loading. *Medical Engineering and Physics*.

[31] Hefzy MS, Yang H (1993). A three-dimensional anatomical model of the human patello-femoral joint, for the determination of patello-femoral motions and contact characteristics. *Journal of Biomedical Engineering*.

[32] Wismans J, Veldpaus F, Janssen J (1980). A three-dimensioal mathematical model of the knee-joint. *Journal of Biomechanics*.

[33] Grood ES, Hefzy MS (1982). An analytical technique for modeling knee joint stiffness—Part I: ligamentous forces. *Journal of Biomechanical Engineering*.

[34] Blankevoort L, Huiskes R (1991). Ligament-bone interaction in a three-dimensional model of the knee. *Journal of Biomechanical Engineering*.

[35] Grood ES, Suntay WJ (1983). A joint coordinate system for the clinical description of three-dimensional motions: application to the knee. *Journal of Biomechanical Engineering*.

[36] Markolf KL, Mensch JS, Amstutz HC (1976). Stiffness and laxity of the knee: the contributions of the supporting structures. A quantitative in vitro study. *Journal of Bone and Joint Surgery*.

[37] Dar FH, Meakin JR, Aspden RM (2002). Statistical methods in finite element analysis. *Journal of Biomechanics*.

[38] Fleming B, Beynnon B, Howe J, McLeod W, Pope M (1992). Effect of tension and placement of a prosthetic anterior cruciate ligament on the anteroposterior laxity of the knee. *Journal of Orthopaedic Research*.

[39] Li G, Zayontz S, Most E, DeFrate LE, Suggs JF, Rubash HE (2004). In situ forces of the anterior and posterior cruciate ligaments in high knee flexion: an in vitro investigation. *Journal of Orthopaedic Research*.

[40] Matsumoto T, Muratsu H, Tsumura N, Mizuno K, Kurosaka M, Kuroda R (2009). Soft tissue balance measurement in posterior-stabilized total knee arthroplasty with a navigation system. *Journal of Arthroplasty*.

